# Relationship between family caregiver burden and physical frailty in older adults without dementia: a systematic review

**DOI:** 10.1186/s13643-017-0447-1

**Published:** 2017-03-14

**Authors:** Thom Ringer, Afeez Abiola Hazzan, Arnav Agarwal, Adam Mutsaers, Alexandra Papaioannou

**Affiliations:** 1 Geriatric Education and Research in Aging Sciences (GERAS), St. Peter’s Hospital, 88 Maplewood Avenue, Hamilton, Ontario L8M 1W9 Canada; 2 Michael G. DeGroote School of Medicine, McMaster University, 1280 Main Street West, Hamilton, Ontario L8S 4L8 Canada; 3 Division of Geriatric Medicine, Department of Medicine, McMaster University, Health Sciences Centre 3W10, 1200 Main Street West, Hamilton, Ontario L8N 3Z5 Canada; 4 University of Toronto Faculty of Medicine, Medical Sciences Building, 1 King’s College Circle #3172, Toronto, Ontario M5S 1A8 Canada

**Keywords:** Frailty, Physical frailty, Care recipient, Older adult, Caregiver, Burden, Community

## Abstract

**Background:**

Physical frailty is a prevalent syndrome in older adults that increases vulnerability for a range of adverse outcomes including increased dependency and death. Caregivers of older adults experience significant physical, emotional, and financial burden, which is associated with poor physical and mental health. While it is known that care recipients’ dementia is associated with burden, the literature regarding the impact of physical frailty on burden has yet to be synthesized. We conducted a systematic review to assess the state of the evidence regarding the relationship between these two prominent concepts in the geriatric literature.

**Method:**

We used a structured search of databases to identify original English-language articles. Two researchers screened the titles and abstracts of all 1202 retrieved studies and then full-text versions of 265 retained studies. Screening was based on a priori inclusion criteria, which included discussion of physical frailty, caregiver burden, and a population of community-dwelling older adults without dementia. Nine included papers underwent data abstraction and critical appraisal using the Cochrane Risk of Bias Tool or the Newcastle-Ottawa Scale (for randomized controlled trials or cross-sectional studies, respectively). Heterogeneity of the included studies precluded meta-analysis.

**Results:**

Five publications had the same author and drew from the same population; these were treated as a single study. Three of our studies were of limited value since they did not include a validated measure of frailty. While caregivers of frail older adults experience burden, the scarce available evidence and lack of studies comparing this population with normative values does not allow conclusions to be drawn about the strength or nature of the relationship. Judging from excluded studies, the term “frailty” is often used without reference to a clear definition or is treated as synonymous with functional impairment or advanced age.

**Conclusions:**

Our review suggests that caregivers of frail older adults experience burden and that the degree of burden may differ from that of other caregiver populations. The limited evidence does not allow conclusions to be drawn or to inform clinical practice. Further research is needed, given the salience of physical frailty and burden.

**Systematic review registration:**

PROSPERO CRD42015019198

**Electronic supplementary material:**

The online version of this article (doi:10.1186/s13643-017-0447-1) contains supplementary material, which is available to authorized users.

## Background

Physical frailty has been defined as “a medical syndrome with multiple causes and contributors that is characterized by diminished strength, diminished endurance, and reduced physiologic function that increases vulnerability for developing increased dependency and/or death” [1]. A recent systematic review involving 15 studies with a total of 44,984 participants found an average pooled prevalence of 9.9% of physical frailty in community-dwelling older adults [2]. Physical frailty has been shown to be a strong predictor of higher mortality risk and reduced life expectancy, as well as increased depression, impairment in activities of daily living, and postoperative complications and hospital length of stay [3–8].

Family caregivers are relatives, partners, friends, or neighbors providing a broad range of assistance for a care recipient and may be primary or secondary caregivers and live with or independently from the recipient [9]. Given the substantial morbidity associated with physical frailty, family caregivers providing care for community-dwelling older adults experience substantial physical, financial, and psychosocial burden [10, 11]. In addition, caregivers of the frail elderly experience financial and emotional strain associated with diminished health-related quality of life and life satisfaction [12].

While caregiver burden associated with community-dwelling older adults has been evidenced, the relationship between physical frailty and family caregiver burden has not been previously synthesized. We conducted a systematic review of the literature to determine whether community-dwelling older adult physical frailty is associated with increased family caregiver burden.

## Methods

**Fig. 1:**
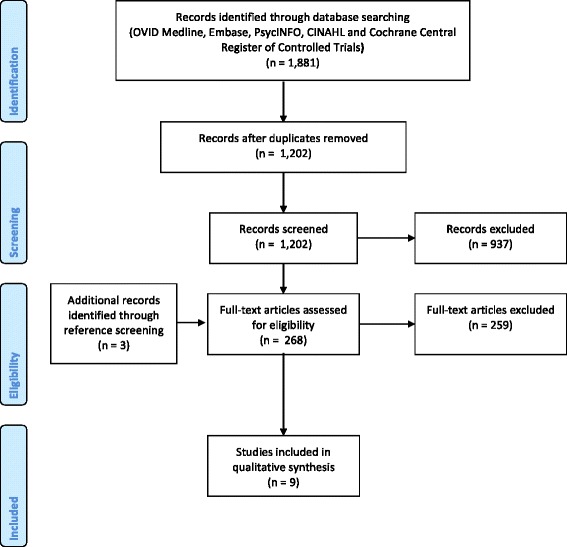
PRISMA flow diagram

We developed and followed a protocol that used the standard systematic review framework [13]. A PRISMA 2009 Flow Diagram is provided as Fig. 1. A completed PRISMA checklist is included as an Additional file 1 with this publication [14].

### Literature search

We conducted a systematic search using Ovid MEDLINE(R) (1946 to present), Embase (1974 to 2015), PsycINFO (1987 to 2015), Cochrane Central Register of Controlled Trials, and CINAHL. The search was conducted by a trained research librarian from inception to April 2015 (Additional file 2). We also emailed three noted scholars in the fields of frailty and/or caregiver burden, who did not identify any additional studies.

### Study selection

Our inclusion criteria were defined a priori. We included English-language studies that included family caregivers of community-dwelling older adults and reported on caregiver burden. Experimental [randomized controlled trial (RCT), controlled trial, quasi-randomized trial, and quasi-experimental], observational (before/after study, prospective cohort study, retrospective cohort study, case-control study, analytic or descriptive cross-sectional study, case report, and case series), and qualitative studies were considered eligible. Systematic reviews addressing our research question were included to identify additional eligible studies. Book chapters, symposium and conference proceedings, essays, editorials, letters, commentaries, narrative reviews, and protocols were excluded.

We defined family caregivers as unpaid caregivers who are typically close relatives (e.g., spouses and children) and provide the bulk of the care and support received by a dependent older adult living in the community (not in a hospital, retirement community, nursing home, or any form of long-term care) [15]. Studies involving solely institutionalized older adults were excluded on the basis that caregiver burden has previously been explored extensively in this setting. Family caregiver burden was defined as any construct or variable representing the physical, psychological, and financial cost of providing care for a loved one [16]. Studies reporting caregiver-related outcomes related to subjective burden (e.g., effects on emotional, social, financial, physical, and spiritual functioning), objective burden (e.g., amount of time spent on caregiving, the caregiving tasks that are performed, and possible financial problems), or both were considered eligible. Studies that employed qualitative measures and quantitative measures (e.g., Caregiver Assessment Tool, Caregiver Burden Screen, and Caregiver Stress Scale) were both included.

Care recipient frailty was defined as any property or syndrome that may relate to physical frailty. Examples of such properties included, but were not limited to, unintentional weight loss, self-reported exhaustion, weakness, slow walking speed, low physical activity, and loss of energy, physical ability, or health. Articles defining frailty solely as a limitation in functional activity were considered ineligible. Finally, studies of older adults with dementia were excluded.

We note that our registered protocol did not include this last exclusion criterion. However, early abstract screening revealed that a large number of studies of caregivers of older adults included only patients with dementia. Based on the known substantial positive association between dementia and caregiver burden and our interest in identifying to the extent possible the impact of physical frailty in particular, we elected to exclude such studies on the assumption that they would be highly confounded [17, 18].

### Study selection

Two investigators (TR, AA) independently screened every original title and abstract identified. Consensus was reached on decisions to advance studies to full text screening, with discrepancies resolved by discussion between the two reviewers and adjudication by a third reviewer (AAH) as necessary. Full text versions of eligible studies were subsequently retrieved for detailed review and were independently screened in duplicate (TR, AM), with discrepancies resolved by discussion and adjudication by a third reviewer (AAH) as necessary. The reference lists of review articles encountered during the full text screening process were examined by both reviewers (TR, AM) to identify additional potentially eligible studies. Title and abstract as well as full text screening were conducted on the DistillerSR platform using pre-designed online forms piloted by a pair of reviewers (TR, AM) and refined to ensure usability.

### Data abstraction and quality assessment

After determining article inclusion, two reviewers (TR, AM) extracted study data into extraction tables in duplicate using piloted and standardized extraction forms and reconciled conflicts by discussion with adjudication by a third reviewer (AAH) as necessary. The following information was extracted from every included study: study design, country, study objectives, general description of care recipient population, care recipient age, care recipient gender, other descriptive information about care recipients (e.g., mini-mental status examination score), general description of caregiver population including age, gender, and additional details (e.g., whether caregivers resided with the recipient; relationship to care recipient). Outcome data regarding how frailty was defined and measured, how family caregiver burden was defined and measured, and reported results regarding the relationship between caregiver burden and physical frailty were extracted into summary tables.

All included papers were critically appraised in duplicate by two reviewers (TR, AM) using piloted and standardized assessment forms, with discrepancies resolved by discussion and adjudication by a third reviewer (AAH) as necessary. Included RCTs were rated on sequence generation, allocation concealment, blinding of participants, personnel and outcome assessors, incomplete outcome data, selective outcome reporting, and other sources of potential bias as per the Cochrane Collaboration’s tool for assessing risk of bias [19]. Included cross-sectional studies were appraised on sample selection, comparability, and outcome assessment based on a published adapted version of the Newcastle-Ottawa Scale [20]. As mentioned below, five papers reported on essentially the same study. However, each paper was appraised separately, since critical appraisal considers not just the analysis and design of a study but the manner in which its results are reported.

We note that our registered protocol had contemplated using the Grading of Recommendations Assessment, Development and Evaluation (GRADE) approach to assess the level of evidence across studies, which posits a systematic and explicit approach to making judgments about the quality of evidence supporting clinical recommendations, such as a practice guideline statement about the effect of a particular intervention [21]. However, factors such as the heterogeneity of designs and outcomes among included studies, and the paucity of interventional studies, precluded the identification of any evidence-based recommendations (regardless of quality), thus rendering GRADE inapplicable.

### Data synthesis

Given significant heterogeneity in study populations, outcomes, and measures across eligible studies, it was not possible to perform a meta-analysis. Accordingly, results were synthesized narratively, with summary tables presenting the key study characteristics, findings, and limitations, and risk of bias assessments associated with each study.

### Role of the funding source

The Geriatric Education and Research in Aging Sciences (GERAS) Centre employed and provided partial funding for the work of two co-authors (AP, AAH) but did not play a role in the selection of the topic, conduct of the study, or interpretation or presentation of the results as an institution acting in a corporate capacity. The GERAS Centre has no financial interest in the outcome of the study.

## Results

A systematic search of five electronic databases identified 1881 original records. Following screening and full-text review, nine eligible articles were identified [22–30]. Five studies were advanced to data abstraction and risk of bias assessment (Fig. 1) [25, 27–30]. The rationale for not advancing all nine of the eligible studies is discussed in the next subsection.

Of the 256 articles excluded after full text review, a large majority (202, 78.9%) did not discuss physical frailty. Some of these studies employed a non-physical definition of frailty, e.g., with reference to Rockwood and colleagues’ Clinical Frail Scale [31]. Other studies defined frailty in terms of impaired functioning, e.g., diminished capacity to perform activities of daily living. Some studies used “frailty” as synonymous with advanced age. In many cases, the term “frail” was used without an explicit definition or a validated tool measuring frailty.

### Characteristics of reviewed studies

Characteristics of the five included studies are presented in Table 1 [25, 27–30]. One study was an RCT [29]. The remainder were cross-sectional in design [25, 27–30].

**Table 1:** Characteristics of included studies

Paper	Country	Design	Description and aims	Population	Care recipient characteristics	Caregiver characteristics
Aggar 2012* [25]	Australia	Cross-sectional	Questionnaire-based longitudinal study. Aim was to compare caregiver reaction, depression, and anxiety in primary family caregivers of older adults enrolled in the control and intervention arms of a trial of an intervention targeting frailty. Timepoints were 3, 6, 9, 12, and 15 months after initiation of the trial.	119 primary informal caregivers of older adults recently discharged from an aged care or rehabilitation service, residing in a major metropolitan area, and participating in a randomized trial targeting frailty. All recipients were frail (FFS ≥3).	Mean age: 84.4±6.0 Female: 71% MMSE>18** Frail; 64.7% Very frail; 35.3%	Mean age; 66.7 ± 13.7 Female; 59.7% Co-residing with CR; 57.1% Self-reported good health; 76% Provide <20 (>40) h/week of care; 52.1%
Comans 2011 [27]	Australia	Cross-sectional	Cross-sectional analysis of baseline characteristics of an older population enrolled in an RCT of community rehabilitation service delivery models. Aim was to identify factors contributing to reduced quality of life and increased caregiver strain (CSI).	107 older adults participating in an RCT of community rehabilitation service delivery models. Participants eligible if referred to a community rehabilitation service for falls or functional decline, ambulatory, nonresident in high-level care, and not unable to participate in a rehabilitation program due to physical or cognitive function. 45 participants had a caregiver who completed a CSI.	Mean age; 78.93 ± 7.67 Female gender; 66% EQ-5D; 0.56 ± 0.31 (reported normative value 0.7) EQ-VAS; 61.76 ± 15.62 (reported normative value 68) FAI; 19.43 ± 8.81 (reported normative value 40.86) AMTS; 8.68 ± 1.19 (reported normative value 8–10) TUG; 20.57 s ± 14.23 s (reported normative value <10) Caregiver available; 42%	(NB; only 42% of participants had caregiver available) Mean CSI; 4.4 ± 3.53
Cullen 1997 [28]	Australia	Australia Cross-sectional	Cross-sectional analysis of population of cognitively impaired older adults drawn from a longitudinal study and their CGs. Aim was to examine associations between CR sociodemographic, caregiver and relationship characteristics with caregiver morbidity singly and after controlling for clinical characteristics of the CRs.	90 dyads consisting of community dwelling older adults with mild or greater levels of cognitive impairment (MMSE <27) and their informal CGs.	Mean age; 79 ± 6 Female gender; 54% Mean MMSE; 23.4 ± 3.6 Married; 54.4%	Mean age; 61 ± 14 Female gender; 81% Spouse of CR; 44% Child/inlaw of CR; 45.6%
Faes 2011 [29]	Netherlands	Randomized controlled trial	RCT of a multifactorial fall prevention program. Aim was to assess whether intervention (program) was more effective than usual geriatric care in preventing falls in frail communitydwelling older fallers, with and without cognitive impairment, and in alleviating subjective caregiver burden in subjects’ CGs.	33 dyads consisting of community dwelling older adults who had fallen at least once in the last 6 months and who met at least 2 of the FFS criteria, and their informal caregivers.	No sociodemographic characteristics (e.g., age) reported. All CRs able to walk at least 15 m independently with or without walking aid. All CRs had life expectancy >12 months. All CRs had MMSE >15.	Mean age (intervention/control); 67.3 ± 13.1/64.3 ± 14.3 Female gender (intervention/control); 50/67% Living with CR (intervention/control); 55/47% Baseline ZBI (intervention/control); 5.2/6.0 Total caregiving hours per week (intervention/control); 8.0/10.5
Kim 2008 [30]	USA	Cross-sectional	Tele-survey-based study comparing caregiving burden and distress, among CGs of 4 types of CRs: cancer, diabetes, dementia, frail older adults.	606 CGs across all 4 groups, including 135 caregivers (“frail elderly” group) of CRs whom their CG described as “frail due to age.”	(“Frail elderly” group only) Mean age; 81.69 ± 9.77	(“Frail elderly” group only) Mean age; 46.23 ± 14.99 Female gender; 47.4% Relationship to CR; Spouse/partner; 0.7% Child/in-law; 50.4% Sibling/in-law; 1.5% Grandchild/in-law; 18.5% Friend/neighbor/nonrelative; 17.8% Other; 11.1%

*This publication represents the index study for a series of five papers by a single set of investigators [22–26]. An explanation of the relationship between these articles appears in the “Results” section of the manuscript

** Care recipient demographics were not reported in this paper [25]; these details were extracted from Aggar 2011b, which involves the same population [24].

Although our review identified nine papers, we have decided that it is most accurate to characterize our review as including five studies. Five of the papers [22–26] had the same principal investigator and involved the same population: caregivers of frail older adults who were enrolled in a RCT of a multifactorial interdisciplinary intervention aimed at reducing mobility-related disability. We note that the study reporting on this RCT was not captured by our database search and would not have met our review’s criteria because the population of the RCT itself did not include caregivers [32]. In a recruitment exercise distinct from the RCT itself, caregivers designated by RCT participants were sent a set of questionnaires.

We contacted the principal investigator on these five publications in December 2016 to clarify the relationships between them. Each paper consists of a different cross-sectional analysis of the data collected through the above-referenced questionnaires. Two of the papers describe cross-sectional analyses conducted at an earlier point in the underlying RCT and involved the caregivers of 93 RCT participants. The remaining three papers describe cross-sectional analyses which were conducted at a later point and involved caregivers of 119 participants in the same RCT. Because each paper reflects a different analysis of essentially the same questionnaire responses from the same population, but was published separately, we have designated one paper as the index study for purposes of extraction and appraisal and refer to it hereinafter as “the Aggar study” [25] and term the remaining publications “the related papers” [22–24, 26].

The largest study had 606 caregiver-care recipient dyads in total, of which 135 included a frail care recipient [30]. The four remaining studies had fewer than 120 dyads [25, 27–30]. The smallest had 33 dyads [29].

Table 2 summarizes key findings from the included studies.

**Table 2:** Summary table of included studies

Paper	Frailty measure	Caregiver burden measure	Summary	Limitations
Aggar 2012* [25]	FFS	CRA HADS	A set of comprehensive, multidisciplinary, and individualized interventions targeting frailty in CRs has some positive effects on CG burden. CGs of CRs in the intervention group reported better health (*F* = 5.303, *p* = 0.023) and selfesteem than the control group (*F* = 4.158, *p* = 0.044). CGs in the intervention group showed continuous improvement in health scores over the duration of the study. Anxiety increased over time significantly in both intervention and control groups (*F* = 2.819, *p* = 0.04). In secondary analysis, CGs who resided with CRs reported significantly higher self-esteem than non-co-resident CGs (*F* = 4.088, *p* = 0.046).	Cross-sectional (single point in time). Risk of survey bias. Outcomes of interest were not part of design of underlying RCT. No subgroup analysis on frail vs. very frail subgroups. Study population is relatively socioeconomically advantaged. Study did not include any non-frail participants for comparison.
Comans 2011 [27]	No direct measure of frailty. Potential proxy measures for components of FFS: TUG, poor balance, use of walking aid (FFS; slow walking speed) Low BMI (<24) and malnutrition (FFS; weight loss)	CSI	In an analysis of a small sample (*N* = 45) of caregiver/care recipient dyads, none of the potential proxy measures for slow walking speed or weight loss was significantly associated with caregiver strain.	Cross-sectional (single point in time). No direct measure of frailty. No subgroup analysis of CRs with and without CGs. Relatively small sample size (45 of 107 CRs had CGs available).
Cullen 1997 [28]	No direct measure of frailty. Potential proxy measures for component of FFS: gait ataxia; extrapyramidal gait disorder (FFS; slow walking speed)	RSS	In caregivers of cognitively impaired older adults, potential proxy measures of CRs’ slow walking speed were significantly associated with irritability and tension. Gait ataxia and extrapyramidal gait disorders were associated with caregiver irritability (*p* < 0.01, z-score = −1.60) and tension (p < 0.05, *z*-score = −1:36), respectively. Neither gait disturbance was significantly associated with CG tiredness, worry, depression, or GHQ scale.	Cross-sectional (single point in time). No direct measure of frailty. Patient population is cognitively impaired; may not be representative of older adults generally. CG burden variables were intercorrelated.
Faes 2011 [29]	All subjects had FFS ≥2. No direct measure of frailty. Potential proxy measures for component of FFS: TUG; walking velocity (FFS; slow walking speed)	ZBI CES-D HADS-A EQ-5D-VAS	In this study of a multifactorial intervention to prevent falls in frail older adults, there was no significant difference between control and intervention groups in potential proxy measures of frailty (TUG or velocity). There was no significant difference in any CG burden measure (including anxiety, depression, and quality of life) between CGs of CRs in the two groups.	No direct measure of frailty. Small study population. Study did not control for cognitive impairment. Study did not include any non-frail participants for comparison (i.e., all participants had FFS ≥2).
Kim 2008 [30]	CGs in the “frail elderly” group reported that they were caring for someone who was “frail due to age.” Frailty not otherwise defined.	Self-reported physical strain, emotional stress, and financial hardship as rated on a 5-point scale (1 = not at all; 5 = great deal/very much). Self-reported amount of money spent on caregiving/mo.	Compared to three other groups of CGs (for patients with cancer, diabetes, and dementia), CGs of CRs whom the CGs themselves described as “frail due to age” reported the least physical strain, emotional stress, and financial hardship. Of the four groups of CGs, carers of frail older adults also spent the least of their own money on caring and less than half the average time on caregiving activities.	Cross-sectional study (single point in time). Risk of survey bias. No direct measure of frailty; relied on CGs’ subjective impression of “frailty due to age,” which may have no relationship with physical frailty.

*This publication represents the index study for a series of five papers by a single set of investigators [22–26]. An explanation of the relationship between these articles appears in the “Results” section of the manuscript

### Care recipient and caregiver populations

The care recipient and caregiver populations included in each study were similar in terms of most descriptive characteristics. Of note, two studies only included care recipients who met certain minimums of cognitive function based on the Mini-Mental Status Examination (MMSE): >18 for the Aggar study [25] and >15 in the RCT [29]. One study only included patients with mild or worse cognitive impairment (MMSE <27) [28]. Among studies reporting on co-residence, approximately half of caregivers co-resided with their care recipient (57.1% [25] in the Aggar study; 47% (control) to 50% (intervention) in the RCT [29]).

### Reporting of care recipient frailty

No study included physical frailty as one of its areas of focus. Only two studies [25, 29] included participants deemed frail using a validated measure (Fried Frail Scale (FFS) [33].

Because they were recruited from an RCT involving a frailty intervention, all participants in the Aggar study were frail (FFS >2) as a criterion for inclusion [25]. These studies did not otherwise report on participants’ frailty. All participants in the included RCT were drawn from a study population previously determined to be frail (FFS ≥2), so recipients’ degree of frailty was not measured as part of the study [29].

Two studies neither explicitly defined or measured frailty qualitatively or quantitatively [27, 28]. Each study, however, included variables that reviewers agreed could be considered potential proxy measures for components of the FFS, such as gait ataxia or extrapyramidal gait disorder [28] which could be considered proxy measures for one of the FFS criteria (slow gait speed), a surrogate indicator for decreased physiologic reserve [33]. Another study analyzed care recipients' “timed up and go”, poor balance, and use of walking aid [27]. In one study, care recipients were deemed frail if their caregiver described them as “frail due to age” [30].

### Reporting of caregiver burden

There was considerable heterogeneity in caregiver burden measures used across included studies. With one exception [30], all studies employed a standardized instrument used to assess caregiver burden. These included the Caregiver Reaction Assessment (CRA), Hospital Anxiety and Depression Scale (HADS), Caregiver Strain Index (CSI), and Relative Stress Scale. One study employed a total of four instruments [29]. Kim et al. asked participants to report their physical strain, emotional stress, and financial hardship on a 5-point scale (1 = not at all; 5 = great deal/very much) [30].

### Burden experienced by caregivers of physically frail older adults

All included studies suggested that caregivers of physically frail older adults experienced negative reactions to caregiving, as well as anxiety and depression. No studies included normative data for purposes of comparison with non-frail populations.

The Aggar study found that caregivers of a population of physically frail older adult care recipients experienced negative reactions to caregiving as measured using the CRA [25]. However, 68% of caregivers found it rewarding to provide care [25].

One study, which directly compared caregivers of frail adults with those of other types of patients, found that caregivers of older adults whom the caregivers themselves described as “frail due to old age” experienced the least physical strain, emotional stress, and financial hardship when compared with caregivers of patients with cancer, diabetes, and dementia [30].

Finally, two studies found that indirect indicators of frailty such as timed up and go, ataxic gait, use of walking aid, poor balance, and weight loss were not associated such measures as caregiver strain, irritability, or tension [28, 29].

### Risk of bias

Risk of bias assessments for the four cross-sectional studies and one RCT are presented in Tables 3 and 4, respectively [19]. Summative risk of bias scores were not calculated for each study, as this practice is disfavoured [19]. The Aggar study drew on a narrow population of study participants from a small geographical region, raising concerns about sample representativeness [25]. Not all of the cross-sectional studies used validated instruments to measure caregiver distress. All included studies relied on self-reported measures of caregiver burden, although this is appropriate given the outcome of interest in these studies was subjective caregiver burden. In our estimation, the one included randomized controlled trial reported incomplete outcome data and did not adequately conceal allocation [29]. As previously discussed, an overall assessment of the quality of the evidence using a tool such as the GRADE approach was deemed inapplicable.

**Table 3:** Critical appraisal of cross-sectional studies using Newcastle-Ottawa Scale

	Selection					Comparability		Outcomes		
	1	2	3	4		1		1	2	
Paper	Representativeness of sample	Sample size	Non-respondents	Ascertainment of exposure	Total Selection Score/5	Comparability of different groups	Total Comparability Score	Assessment of outcome	Statistical test	Total Outcomes Score/2
Aggar 2012* [25]	(c)	(a)*	(a)*	(a)**	4	(a)*	1	(c)	(a)*	1
Comans 2011* [27]	(c)	(b)	(c)	(a)**	2	(a)*	1	(c)	(a)*	1
Cullen 1997 [28]	(b)*	(b)	(a)*	(a)**	4	(a)*	1	(c)	(a)*	1
Kim 2008 [30]	(a)*	(a)*	(c)	(b)	2	(a)*	1	(c)	(a)*	1

*This publication represents the index study for a series of five papers by a single set of investigators [22–26]. As appraisal concerns not just study design and execution but also the way results are presented, it is based on a composite of these five papers. An explanation of the relationship between these articles appears in the “Results” section of the manuscript.

Selection

1. Representativeness of the sample

(a) Representative of the average in the target population (all subjects or random sampling) (*); (b) Somewhat representative (non-random sampling) (*); (c) Selected group of users; (d) Insufficient information

2. Sample size

(a) Justified and satisfactory (*); (b) Not justified

3. Non-respondents

(a) Comparability between respondents’ & nonrespondents’ characteristics established + response rate satisfactory (*); (b) Response rate unsatisfactory or comparability is unsatisfactory; (c) Insufficient information

4. Ascertainment of exposure (risk factor)

(a) Validated measurement tool (**); (b) Tool is described but is non-validated; (c) Insufficient information

Comparability

1. Subjects in different groups are comparable based on study design or analysis, and confounding factors are controlled

(a) Study controls for most important factor (*); (b) Study controls for any additional factor (*); (c) Insufficient information

Outcome

1. Assessment of outcome

(a) Independent blind assessment (**); (b) Record linkage (**); (c) Self-report (*); (d) Insufficient information

2. Statistical test

(a) Test used to analyze data is clearly described is appropriate, and measurement of association is presented including confidence intervals & probability (*); (b) Test not appropriate, not described, or incomplete

**Table 4:** Critical appraisal of RCT using Cochrane Collaboration guidelines

	A	B	C	D	E	F
Study	Adequate sequence generation	Adequate allocation concealment	Adequate blinding	Incomplete outcome data addressed	Free of selective reporting	Free of other bias
Faes 2011 [29]	+	−	+	−	+	+

A - Did the allocation sequence involve a random component or minimization?

B - Was allocation adequately concealed, e.g., through central allocation?

C - Were participants and key study personnel reliably blinded and/or it was unlikely that outcome measurement would be influenced by lack of blinding?

D - Was incomplete outcome data adequately addressed?

E - Are reports of the study free of suggestions of selective reporting, e.g., the protocol is available and all outcomes were reported in a prespecified way?

F - Was the study apparently free of other problems that could put it at a risk of bias?

## Discussion

Research has explored associations between caregiver burden and several classic geriatric syndromes such as dementia, functional decline, multimorbidity, falls, and geriatric depression [17, 34–37]. Frailty is increasingly being recognized as a true “geriatric giant” in its own right. Yet, it differs from these syndromes in that it offers a highly complex framework for understanding the relationships between physiologic reserve and a wide range of outcomes. Its clinical applicability is also attracting greater attention.

While a purely physical understanding of frailty has conceptual limitations, even those who espouse a frailty construct based on accumulated deficits and psychosocial factors recognize that strength, speed, and body mass are essential components of physiologic reserve [38]. Our review represents the first effort to synthesize evidence regarding the association between this clinically and academically significant phenomenon in community-dwelling older adults receiving care and degree of burden experienced by family caregivers.

Our review suggests that caregivers of physically frail older adults experience depression, anxiety, and other negative effects of caregiving. Though not included in abstraction or appraisal for this review, it bears mention that several of the related papers by Aggar and colleagues also reported that caregivers experienced anxiety and depression, with as much as 15% experiencing borderline depression (HADS-D 8-10) and as much as 10% experiencing abnormal depression (HADS-D ≥11) [22]. Up to 24% experienced borderline anxiety (HADS-A 8-10), while 12% experienced abnormal anxiety (HADS-A ≥11) [22].

Another study also concluded that, compared with caregivers of other types of care recipients, caregivers of patients whom they subjectively believed were frail due to old age appeared to experience less burden on a number of dimensions, including hours spent caring and financial burden [30]. However, the absence of normative data in the included studies did not allow us to conclude whether prevalence of depression and anxiety is higher or lower than in the general population.

Finally, though not included for abstraction and appraisal, one of the related papers by Aggar and colleagues explicitly explored the association between frailty and caregiver burden, finding that caregivers of care recipients deemed severely frail (FFS >3) did not differ from caregivers of merely frail (FFS = 3) in terms of their responses regarding both negative aspects of caregiving (e.g., time demands) and positive aspects (e.g., self-esteem), though the significance of this relationship was not stated [24].

Although results were based on a small number of studies with some risk of bias, studies did surface findings of interest that emphasize a need for further research in this area. First, some caregivers of frail older adults find it rewarding to provide care [23, 25]. Second, compared with caregivers of care recipients with morbid medical conditions, caregivers of patients who were frail due to old age appeared to experience less burden on a number of dimensions, including hours spent caring, and financial burden [30]. Both of these findings serve to underscore the importance of a nuanced understanding of the richness and complexity of the experience of caring for frail older adults in particular.

We note that while the manuscript was undergoing review, three of the authors (TR, AAH, and AP) published a cross-sectional study examining the relationship between caregiver burden and physical frailty, which found that frailty was independently associated with burden in a convenience sample of 45 community-dwelling caregiver-care recipient dyads [39].

### Limitations

Our study used well-defined a priori criteria and a rigorous systematic methodology. We do note three potential limitations. First, non-English-language studies were excluded. Second, the relatively small number of included papers, and the fact that five publications consisted of different analyses of essentially the same data and were treated as papers related to a single index study, may impact generalizability of findings and speaks to a substantial gap in the evidence. Finally, the findings are limited by the paucity of normative comparisons in the included studies.

## Conclusions

Our review suggests that few, if any, studies have yet evaluated the relationship between physical frailty and caregiver burden as their primary objective. Despite broad criteria for both frailty and burden, our review yielded a relatively small number of eligible studies. That being said, the diversity of research questions and approaches represented in our review reveals that a range of interesting opportunities exist at the interface of these two highly salient phenomena.

Our review of the evidence also identified numerous potential methodological improvements to inform future projects. First, measures and definitions of “frailty” were heterogeneous among included and excluded studies. A large number defines it solely in functional terms, as synonymous with old age, or use it without any definition at all. This reflects a lack of consensus in the frailty literature generally [40].

Second, few studies specifically considered positive attributes of caregiving (emotional, spiritual, physical, or other) or the factors, such as psychological resilience, which allow certain caregivers to avoid the negative impacts of strain [41]. Further, none of the included studies examined outcomes representing the effect of increased burden on quality of care experienced by the patient population. Future studies, particularly those of an interventional nature, might benefit from addressing these limitations.

## Additional files

Additional file 1: Search strategy. (DOC 23 kb)
Additional file 2: PRISMA Checklist. (DOC 62 kb)

## Abbreviations

AMTS: Abbreviated mental test score
BMI: Body mass index
CES-D: Center for Epidemiologic Studies Depression Scale
CG: Caregiver
CR: Care recipient
CRA: Caregiver Reaction Assessment
CSI: Caregiver Strain Index
EQ-5D-VAS: EQ-5D visual analog scale
FFS: Fried Frail Scale
HADS: Hospital Anxiety & Depression Scale
HADS-A: Hospital Anxiety & Depression Scale - Anxiety subscale
RSS: Relatives’ Strain Scale
TUG: Timed up and go
ZBI: Zarit Burden Interview

## References

[1] Morley JE, Vellas B, van Kan GA, Anker SD, Bauer JM, Bernabei R, Cesari M, Chumlea WC, Doehner W, Evans J, Fried LP, Guralnik JM, Katz PR, Malmstrom TK, McCarter RJ, Gutierrez Robledo LM, Rockwood K, von Haehling S, Vandewoude MF, Walston J (2013). Frailty consensus: a call to action. J Am Med Dir Assoc.

[2] Collard RM, Boter H, Schoevers RA, Oude Voshaar RC (2012). Prevalence of frailty in community-dwelling older persons: a systematic review. J Am Geriatr Soc.

[3] Shamliyan T, Talley KMC, Ramakrishnan R, Kane RL (2013). Association of frailty with survival: a systematic literature review. Ageing Res Rev.

[4] Feng L, Nyunt MS, Feng L, Yap KB, Ng TP (2014). Frailty predicts new and persistent depressive symptoms among community-dwelling older adults: findings from Singapore longitudinal aging study. J Am Med Dir Assoc.

[5] Garre-Olmo J, Calvo-Perxas L, Lopez-Pousa S, De Gracia BM, Vilalta-Franch J (2013). Prevalence of frailty phenotypes and risk of mortality in a community-dwelling elderly cohort. Age Ageing.

[6] De La Rica-Escuin M, Gonzalez-Vaca J, Varela-Perez R, Arjonilla-Garcia MD, Silva-Iglesias Mm Oliver-Carbonell JL, Abizanda P. Frailty and mortality or incident disability in institutionalized older adults: The FINAL Study. Maturitas. 2014;78(4):329-34. 10.1016/j.maturitas.2014.05.022 24929996

[7] Vermeulen J, Neyens JCL, van Rossum E, Spreeuwenberg MD, de Witte LP (2011). Predicting ADL disability in community-dwelling elderly people using physical frailty indicators: a systematic review. BMC Geriatr.

[8] Makary MA, Segev DL, Pronovost PJ, Syin D, Bandeen-Roche K, Patel P, Takenaga R, Devgan L, Holzmueller CG, Tian J, Fried LP (2010). Frailty as a predictor of surgical outcomes in older patients. J Am Coll Surg.

[9] Family Caregiver Alliance National Center on Caregiving. Definitions. 31 Jan 2014. Available at https://www.caregiver.org/pilotIntegration/indexPersistent.html?uri=/definitions-0. Accessed 1 Mar 2017.

[10] Garlo K, O’Leary JR, Van Ness PH, Fried TR (2010). Caregiver burden in caregivers of older adults with advanced illness. J Am Geriatr Soc.

[11] Schulz R, Sherwood PR (2008). Physical and mental effects of family caregiving. Am J Nurs.

[12] Dahlrup B, Ekstrom H, Nordell E, Elmstahl S (2015). Coping as a caregiver: a question of strain and its consequences on life satisfaction and health-related quality of life. Arch Geront Geriatr.

[13] Moher D, Shamseer L, Clarke M, Ghersi D, Liberati A, Petticrew M (2015). Preferred reporting items for systematic review and meta-analysis protocols (PRISMA-P) 2015 statement. Syst Rev.

[14] Shamseer L, Moher D, Clarke M, Ghersi D, Liberati A, Petticrew M, Shekelle P, Stewart L, PRISMA-P Group (2015). Preferred reporting items for systematic review and meta-analysis protocols (PRISMA-P) 2015: elaboration and explanation. BMJ.

[15] Candy B, Jones L, Drake R, Leurent B, King M. Interventions for supporting informal caregivers of patients in the terminal phase of a disease. Cochrane Database Syst Rev. 2011;(6):CD007617. 10.1002/14651858.CD007617.pub2 PMC13247825 21678368

[16] Zarit SH, Todd PA, Zarit JM (1986). Subjective burden of husbands and wives as caregivers: a longitudinal study. Gerontologist.

[17] Chiao CY, Wu HS, Hsiao CY (2015). Caregiver burden for informal caregivers of patients with dementia: a systematic review. Int Nurs Rev.

[18] Wolfs CA, Kessels A, Severens JL, Brouwer W, de Vugt ME, Verhey FR, Dirksen CD (2012). Predictive factors for the objective burden of informal care in people with dementia: a systematic review. Alzheimer Dis Assoc Disord.

[19] Higgins JPT, Green S, eds. Cochrane handbook for systematic reviews of interventions Vers 5.1.0. Cochrane Collaboration. 2011. Available at http://training.cochrane.org/handbook. Accessed 1 Mar 2017.

[20] Wells GA, Shea B, O’Connell D, Peterson J, Welch V, Losos M, Tugwell P, Ottawa Hospital Research Institute. The Newcastle-Ottawa Scale (NOS) for assessing the quality of nonrandomised studies in meta-analyses. Available from http://www.ohri.ca/programs/clinical_epidemiology/oxford.asp. Accessed 1 Mar 2017.

[21] GRADE Working Group (2004). Grading quality of evidence and strength of recommendations. Brit Med J.

[22] Aggar C, Ronaldson S, Cameron ID (2010). Reactions to caregiving of frail, older persons predict depression. Int J Mental Health Nurs.

[23] Aggar C, Ronaldson S, Cameron ID (2011). Reactions to caregiving in frailty research. Arch Geront Geriatr.

[24] Aggar C, Ronaldson S, Cameron ID (2011). Self-esteem in carers of frail older people: resentment predicts anxiety and depression. Aging Ment Health.

[25] Aggar C, Ronaldson S, Cameron ID. Reactions to caregiving during an intervention targeting frailty in community living older people. BMC Geriatr. 25;12(66). DOI: 10.1186/1471-2318-12-66. 10.1186/1471-2318-12-66 PMC3571864 23095644

[26] Aggar C, Ronaldson S (2014). Residential respite care is associated with family carers experiencing financial strain. Australasian J Ageing.

[27] Comans TA, Currin ML, Brauer SG, Haines TP (2011). Factors associated with quality of life and caregiver strain amongst frail older adults referred to a community rehabilitation service: implications for service delivery. Disability Rehab.

[28] Cullen JS, Grayson DA, Jorm AF (1997). Clinical diagnoses and disability of cognitively impaired older persons as predictors of stress in their carers. Int J Geriatr Psych.

[29] Faes MC, Reelick MF, Melis RJ, Borm GF, Esselink RA, Rikkert MGO (2011). Multifactorial fall prevention for pairs of frail community-dwelling older fallers and their informal caregivers: a dead end for complex interventions in the frailest fallers. J Am Med Dir Assoc.

[30] Kim Y, Schulz RJ (2008). Family caregivers’ strains: comparative analysis of cancer caregiving with dementia, diabetes, and frail elderly caregiving. Aging Health.

[31] Rockwood K, Song X, MacKnight C, Bergman H, Hogan DB, McDowell I, Mitnitski A (2005). A global clinical measure of fitness and frailty in elderly people. Can Med Assoc J.

[32] Fairhall N, Sherrington C, Kurrle SE, Lord SR, Lockwood K, Cameron ID (2012). Effect of a multifactorial interdisciplinary intervention on mobility-related disability in frail older people: randomised controlled trial. BMC Med.

[33] Fried LP, Tangen CM, Newman AB, Hirsch C, Gottdiener J, Seeman T, Tracy R, Kop WJ, Burke G, McBurnie MA (2001). Frailty in older adults: evidence for a phenotype. J Gerontol A Biol Sci Med Sci.

[34] Covinsky KE, Newcomer R, Fox P, Wood J, Sands L, Dane K, Yaffe K (2003). Patient and caregiver characteristics associated with depression in caregivers of patients with dementia. J Gen Intern Med.

[35] Giovannetti ER, Wolff JL, Xue QL, Weiss CO, Leff B, Boult C, Hughes T, Boyd CM (2012). Difficulty assisting with health care tasks among caregivers of multimorbid older adults. J Gen Intern Med.

[36] Kuzuya M, Masuda Y, Hirakawa Y, Iwata M, Enoki H, Hasegawa J, Izawa S, Iguchi A (2006). Falls of the elderly are associated with burden of caregivers in the community. Int J Geriatr Psychiatry.

[37] Kang HS, Myung W, Na DL, Kim SY, Lee JH, Han SH, Choi SH, Kim S, Kim S, Kim DK (2014). Factors associated with caregiver burden in patients with Alzheimer’s disease. Psychiatry Investig.

[38] Crone P, Lally F (2011). Frailty: joining the giants. Can Med Assoc J.

[39] Ringer T, Hazzan AA, Kennedy C, Karampatos S, Patterson C, Marr S, Misiaszek B, Woo T, Ioannidis G, Papaioannou A (2016). Care recipients’ physical frailty is independently associated with subjective burden in informal caregivers in the community setting: a cross-sectional study. BMC Geriatr.

[40] Op het Veld LP, van Rossum E, Kempen GI, de Vet HC, Hajema K, Beurskens AJ (2015). Fried phenotype of frailty: cross-sectional comparison of three frailty stages on various health domains. BMC Geriatr.

[41] Harmell AL, Chattillion EA, Roepke SK, Mausbach BT (2011). A review of the psychobiology of dementia caregiving: a focus on resilience factors. Current Psychiatry Reports.

[42] Ringer T, Hazzan AA, Agarwal A, Mutsaers A, Papaioannou A. Relationship between physical frailty in community dwelling older adults and informal caregiver burden: a systematic review. Abstracts from the 36th Annual Scientific Meeting of the Canadian Geriatrics Society, Vancouver BC. April 16, 2016. Can Geriatr J. 2016;19(3):129.

