# Supplementary material for: Relationship between family caregiver burden and physical frailty in older adults without dementia: a systematic review
Source: Syst Rev. 2017 Mar 14;6:55. doi: 10.1186/s13643-017-0447-1 (PMC5351063; doi:10.1186/s13643-017-0447-1)
Supplement: Additional file 1: — Search strategy. (DOC 23 kb) [file 13643_2017_447_MOESM1_ESM.doc]

**Additional file 1: Database search terms**

1. exp Aged/

2. older*.mp.

3. elderly.mp.

4. 1 or 2 or 3

5. community.mp.

6. home.mp.

7. house.mp.

8. apartment.mp.

9. condo*.mp.

10. 5 or 6 or 7 or 8 or 9

11. 4 and 10

12. Family Nursing/

13. (family adj2 caregiv*).ti.

14. exp Caregivers/

15. ((informal or family or spous* or husband or wife) adj3 (care* or caregiv*)).tw.

16. 12 or 13 or 14 or 15

17. frail*.mp.

18. 11 and 16 and 17

19. limit 18 to (biography or case reports or comment or editorial or interview or letter)

20. 18 not 19
